# Associations of maternal and paternal antenatal mood with offspring anxiety disorder at age 18 years

**DOI:** 10.1016/j.jad.2015.08.012

**Published:** 2015-11-15

**Authors:** Lauren E. Capron, Vivette Glover, Rebecca M. Pearson, Jonathan Evans, Thomas G. O’Connor, Alan Stein, Susannah E. Murphy, Paul G. Ramchandani

**Affiliations:** a Centre of Mental Health, Imperial College London, London, UK; b IRDB, Imperial College London, London, UK; c Academic Unit of Psychiatry, University of Bristol, Bristol, UK; d Wynne Center for Family Research and Department of Psychiatry, University of Rochester Medical Center, New York, NY, USA; e Department of Psychiatry, University of Oxford, Oxford, UK

**Keywords:** (EPDS), Edinburgh Postnatal Depression Scale, (CCEI), Crown Crisp Experiential Index, (CIS-R), Clinical Interview Schedule – Revised, (ALSPAC study), Avon Longitudinal Study of Parents and Children, Depression, Anxiety, Foetal programming, Maternal, Paternal, ALSPAC

## Abstract

**Objective:**

Maternal antenatal depression and anxiety are associated with increased risk of childhood behavioural and emotional problems in offspring; it remains unclear to what extent this is due to a maternal biological impact on foetal development. Here, we compare associations between maternal and paternal antenatal depression and anxiety with offspring anxiety disorders, thus controlling for some genetic and shared environmental factors.

**Methods:**

We used data from the ALSPAC population cohort including measures of antenatal parental depression and anxiety. At 18 years, offspring completed the CIS-R interview, yielding diagnoses for anxiety disorders. Results were adjusted for confounding variables including parental postnatal depression and anxiety.

**Results:**

Children of women with antenatal depression (18 weeks gestation), had an increased risk of anxiety disorders at 18 years of age (11.1% vs. 6.2%; adj. OR 1.75 (1.19, 2.58); *p*=0.01). Children of women with antenatal anxiety had increased risk of co-morbid anxiety and depression (adj. OR 1.39 (1.06, 1.82); *p*=0.02). No such associations were found with paternal antenatal depression or anxiety.

**Limitations:**

There was a high attrition rate from the original cohort to the CIS-R completion at 18 years postpartum. Parental mood was only assessed together at one time point during the antenatal period.

**Conclusions:**

The differences in the association between maternal and paternal mood during pregnancy and child outcomes supports the hypothesis that foetal programming may account, at least in part, for this association. We highlight the potential opportunity for preventative intervention by optimising antenatal mental health.

## Highlights

• We compare parental antenatal depression and anxiety with offspring anxiety disorder.
• Maternal antenatal depression was associated with increased risk of offspring anxiety.
• No such associations were found with paternal antenatal depression or anxiety.
• Highlights opportunity to improve offspring mental health via preventive intervention.

## 1 Introduction

There is accumulating evidence that maternal antenatal stress, including depression and anxiety, is associated with an increased risk of behavioural and emotional problems in the child (Glover, 2014). Changes to the in utero environment during pregnancy could result in an altered developmental trajectory of the foetus and child; this is known as foetal programming (Barker et al., 2013; Gluckman and Hanson, 2004; Jansson and Powell, 2007). This hypothesis proposes that if a particular stimulus or insult occurs during any sensitive period of in utero development it can result in alterations to the development of the foetus, leading to an increase in the risk of disease later on in life (Lucas et al., 1999). Maternal anxiety and depression are potential exposures that may alter long-term child development in this way. Many studies have shown maternal depression and anxiety during pregnancy to be associated with altered infant temperament and behavioural stress reactivity (Buitelaar et al., 2003; Davis and Sandman, 2010; Monk et al., 2012). These altered infant responses can be an indicator of vulnerability for the development of psychopathologies later in life (Schlotz and Phillips, 2009). There are also a small number of studies that show an increase in psychiatric risk during later childhood and adolescence (Pawlby et al., 2011; Pearson et al., 2013a). However, it is important to consider that although there are associations, these do not necessarily infer causality. Some researchers have argued that these findings may be the result of residual confounding variables such as birth weight and social economic status (Davey Smith and Ebrahim, 2001). Confounders are an issue in all epidemiological studies, but are of particular concern in foetal programming studies, as confounding factors may be inadequately measured or omitted, and this residual confounding may lead to false positive findings (Davey Smith, 2008).

One way of disentangling this issue is to compare the strength of associations between an exposure among mothers and offspring outcomes, and the same exposure among fathers and offspring outcomes (Davey Smith, 2008). The foetal programming hypothesis would propose that if there is a direct biological effect of maternal mental health on foetal development and subsequent offspring outcome, then the association between antenatal stress exposure and offspring should be stronger in mothers than in fathers. Comparatively, a stronger or equivalent association with fathers may be indicative of genetic or other environmental mechanisms or an effect of unmeasured residual confounders. Lewis et al. (2013) reviewed different approaches for assessing the strengths of causal inference regarding antenatal risk factors for childhood behavioural and psychiatric disorders and commented that the use of maternal versus paternal comparisons is a means of increasing causal inference by approximating a quasi-experimental design.

To date there are relatively few studies focussing on the impact of paternal mental health during the perinatal period on the subsequent development of the child (Ramchandani and Psychogiou, 2009). Furthermore, studies looking at antenatal depression and/or anxiety in mothers and fathers and the effects on the offspring have usually been assessed as separate entities. One study, which compared the associations between maternal and paternal depression and anxiety states during pregnancy with later child outcome, examined early childhood outcomes (Van Batenburg-Eddes et al., 2013). The authors compared associations of parental depression and anxiety during pregnancy with attention problems in early childhood using data from two large cohort studies; ALSPAC and Generation R. This study produced conflicting finding; as the Generation R cohort demonstrated no increased association with maternal or paternal stress, whereas the ALSPAC cohort showed an association between maternal depression and anxiety during pregnancy with the onset of attention problems in early childhood but not with paternal antenatal depression and anxiety (Van Batenburg-Eddes et al., 2013). A recent study by O’Donnell et al. (2014a) compared the effects of antenatal maternal and paternal stress on offspring psychopathology at 13 years of age. Results indicated that maternal antenatal anxiety and depression predicted lasting effects on offspring behavioural and emotional problems, with no equivalent effect with fathers. However, the authors only followed the children into early adolescence and used the Strengths and Difficulties Questionnaires to measure offspring behaviour, which is not a diagnostic tool. Another recent paper by Pearson et al. (2013b) demonstrated that maternal anxiety and depression in pregnancy had an association with offspring depression at 18 years but did not look at the possible associations with offspring anxiety.

This study therefore aims to extend pre-existing work by comparing the associations between maternal and paternal antenatal depression and anxiety and offspring anxiety at 18 years of age, assessed by a structured psychiatric interview. The focus lay on anxiety because anxiety disorders remain one of the most common and impairing forms of psychopathology in late adolescence, but are relatively understudied in comparison to other disorders, such as depression. As of 2010, 4.5% of the global population (273 million people) suffer from an anxiety disorder (Blair Simpson et al., 2010). If foetal programming effects are occurring we would expect exposure to maternal depression and anxiety in utero to result in an increased risk of offspring anxiety disorders compared with paternal exposure. Conversely, similar outcomes for maternal and paternal depression and anxiety exposure would be indicative of other mechanisms, such as genetic continuity, other environmental or unmeasured confounding effects.

## 2 Methods

### 2.1 Participants

The current study used data from the Avon Longitudinal Study of Parents and Children (ALSPAC Study); a large longitudinal birth cohort study based in and around Bristol, UK. All women resident within the defined geographical area with an expected date of delivery between 1 April 1991 and 31 December 1992 were eligible for inclusion. ALSPAC recruited 14,541 pregnant women during this time-period. These pregnancies resulted in 14,062 live births and 13,988 children at 1 year of age. Recruitment is detailed in previous publications describing the ALSPAC Study (Boyd et al., 2012; Golding et al., 2001). The current study uses data from the offspring sample (singletons only) completing the assessment at 18 years of age (*n*=4303). The study website contains details of all the data that are available through a fully searchable data dictionary (http://www.bris.ac.uk/alspac/researchers/data-access/data-dictionary/). Measures of antenatal depression and anxiety were collected at 18 weeks gestation. This was the only time point during the antenatal period where these were measured in both mothers and fathers.

## 3 Measures

### 3.1 Parental depression

Depression in mothers and fathers was measured using the Edinburgh Postnatal Depression Scale (EPDS; (Cox et al., 1987)) at 18 weeks gestation. This widely used questionnaire is designed to screen for self-reported depressive symptoms during and after pregnancy and has been validated for use in both men and women (Matthey et al., 2001; Murray and Cooper, 1997). A cut-off point of ≥13 was used to indicate probable depression as this is a standardised cut off for women and is often used in studies examining paternal depression (Fisher et al., 2012; Korhonen et al., 2012; Meltzer-Brody et al., 2013; Ramchandani et al., 2008)

### 3.2 Parental anxiety

Parental anxiety was measured using the Crown Crisp Experiential Index (CCEI) at 18 weeks gestation, which is a validated self-rating inventory (Birtchnell et al., 1988). A cut-off of ≥8 was selected to identify the highest scoring 15% in the maternal anxiety data, consistent with previous studies (Glover et al., 2004; Heron et al., 2004). Paternal anxiety was scored using the same cut off as maternal anxiety for comparability (CCEI≥8).

### 3.3 Offspring anxiety

At 18 years of age the offspring completed the Clinical Interview Schedule Revised (CIS-R) (Lewis et al., 1992). The interview was a self-administered, computerised interview completed at the research clinic (Stringaris et al., 2014). This interview gives an ICD-10 diagnosis and severity scores for the symptoms of depression and anxiety and other common psychiatric disorders (Pearson et al., 2013a). This study primarily investigated offspring with a pure anxiety phenotype, which includes generalised anxiety disorder, social phobia, specific phobia, agoraphobia and panic disorder and excluded those with comorbid depression. A binary variable indicated a diagnosis of an anxiety disorder on the CIS-R or no diagnosis was used as the outcome measure.

### 3.4 Data analysis

The statistical analyses were conducted in four steps: (1) Descriptive analyses were firstly explored for all key variables. (2) Associations between parental depression and anxiety and the risk of offspring anxiety were then assessed using logistic regression analyses, computing odds ratios as a measure of risk. (3) Results were then adjusted for key confounding variables; maternal age, gestational age, birth weight, parity, maternal alcohol consumption during pregnancy, maternal smoking during pregnancy, maternal educational status and postnatal parental depression and anxiety (measured at 8 weeks postpartum) through the use of multivariate analysis. (4) Multivariate analyses were repeated, with the outcome now including all those who had been diagnosed with anxiety (including those with comorbid depression).

## 4 Results

### 4.1 Demographics

Table 1 shows the demographic characteristics of the whole ALSPAC population sample and the sub-set of participants who undertook the CIS-R assessment at age 18 years. There are some differences in maternal age, percentage of male offspring, maternal antenatal smoking (average number of cigarettes per day), parity, maternal educational status, maternal antenatal and postnatal depression and anxiety, as well as paternal antenatal depression; however, their magnitude was relatively small. On average, the participants remaining in this study at age 18 years had significantly older mothers with higher educational attainment and overall lower levels of parental depression.

When examining the percentage of parents with antenatal depression and anxiety in the subset of participants who undertook the CIS-R, 418 mothers (10.7%) and 114 fathers (3.3%) reported symptoms of antenatal depression (EPDS score ≥13); 725 mothers (16.8%) and 238 fathers (6.9%) reported symptoms of antenatal anxiety (CCEI-Anxiety subscale score ≥8). These scores are similar to other studies that have measured parental depression and anxiety during the antenatal period, with meta-analyses reporting widely varying rates of anxiety in the perinatal period (Goodman et al., 2014) and rates of diagnosed depression of 5% in men in the perinatal period and 10–13% in women (Paulson and Bazemore, 2010).

### 4.2 Associations between parental depression and anxiety and offspring diagnosis of anxiety

At 18 years of age 4303 participants undertook the CIS-R, 29.3% of the original cohort. This sub-set of participants are those who remained in the ALSPAC study at 18 years of age and returned to complete the assessment. The demographic characteristics of this subset compared to the total study population are displayed in Table 1. Of these 283 (7.2%) met criteria for pure anxiety (an anxiety disorder with no depressive comorbidity). Children of mothers with high depressive symptoms (EPDS score ≥13) at 18 weeks gestation had an increased risk of anxiety diagnosis at 18 (11.1% vs. 6.2%; OR 1.71(1.20, 2.44); *p*<0.01; *n*=3599). The same increased risk was evident for offspring anxiety at 18 years for mothers reporting high levels of antenatal anxiety (CCEI-Anxiety subscale score ≥8) at 18 weeks gestation (9.2% vs. 6.6%; OR 1.43 (1.06, 1.94); *p*=0.02; *n*=3537). This association was not evident between paternal antenatal mood and offspring anxiety at 18 years (Paternal depression: 7.6% vs. 6.7%; OR 1.14 (0.55, 2.38); *p*=0.73; *n*=3187; paternal anxiety: 8.9% vs. 6.6%; OR 1.39 (0.85, 2.27); *p*=0.19; *n*=3181).

### 4.3 Multivariate analyses

**Fig. 1:**
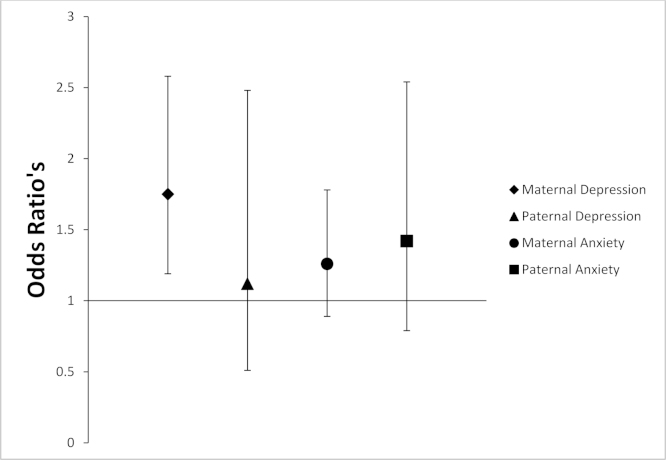
Maternal and paternal prenatal depression and anxiety symptoms and offspring anxiety at 18 years of age. Data is represented as odds ratios±confidence intervals. Groups: maternal depression (EPDS score ≥13), paternal depression (EPDS score ≥13), maternal anxiety (CCEI-Anxiety subscale score ≥8), paternal anxiety (CCEI-Anxiety subscale score ≥8).

The above analyses were re-conducted adjusting for possible confounding variables; maternal age, gestational age, birth weight, parity, maternal alcohol consumption during pregnancy, maternal smoking during pregnancy, maternal educational status and parental postnatal mood measured at 8 weeks postnatally. Mothers with high depressive symptoms at 18 weeks gestation had an increase in the risk of their child being diagnosed with anxiety (adj. OR 1.75 (1.19, 2.58); *p*=0.01; *n*=3395). However, the association between maternal antenatal anxiety and offspring anxiety at 18 years attenuated (adj. OR 1.26 (0.89, 1.78); *p*=0.19; *n*=3361). Fathers with high levels of depressive or anxiety symptoms at 18 weeks gestation did not have an increased risk of their child being diagnosed with anxiety (Paternal depression: adj. OR 1.12 (0.51, 2.48); *p*=0.77; *n*=3025; Paternal anxiety: adj. OR 1.42 (0.79, 2.54); *p*=0.24; *n*=3018) (Table 2; Fig. 1).

These findings were also adjusted for a later postnatal time point for parental mood (21 months postpartum). Mothers with high levels of depressive symptoms at 18 weeks were more likely to have a child with anxiety (adj. OR 1.62 (1.06, 2.47); *p*=0.02; *n*=3129) after adjusting for confounding variables including postnatal mood at 21 months postpartum. Conversely, there was no association between maternal anxiety (adj. OR 1.26 (0.89, 1.78); *p*=0.19; *n*=3361), paternal anxiety (adj. OR 1.73 (0.89, 3.37); *p*=0.11; *n*=2190) or paternal depression (adj. OR 0.84 (0.25, 2.85); *p*=0.78; *n*=2209) after adjusting for confounding variables including postnatal mood at 21 months postpartum.

Parental depression and anxiety scores were also assessed as continuous variables as predictors of offspring anxiety. There was an association between the level of maternal antenatal depression (adj. OR 1.05 (1.02, 1.08); *p*<0.01; *n*=3395) and anxiety symptoms (adj. OR 1.07 (1.03, 1.11); *p*<0.01; *n*=3361) with offspring anxiety disorders at 18 years but no association between paternal antenatal depression (adj. OR 1.02 (0.98, 1.06); *p*=0.35; *n*=3025) or anxiety (adj. OR 1.03 (0.97, 1.10); *p*=0.33; *n*=3018) and offspring anxiety disorders.

### 4.4 Associations between parental depression and anxiety and offspring diagnosis of anxiety (including those comorbid for depression)

All analyses were then repeated, including all offspring reporting anxiety, including those with a co-morbid diagnosis of depression. All results were adjusted for the same confounding variables as in the previous analyses and postnatal mood (8 weeks postpartum). Mothers with an EPDS score ≥13 at 18 weeks gestation had an increase in the risk of their child being diagnosed with anxiety (adj. OR 1.63 (1.19, 2.25); *p*<0.01; 3681). Mothers with a CCEI-Anxiety subscale score ≥8 at 18 weeks gestation had an increase in the risk of their child being diagnosed with anxiety (adj. OR 1.39 (1.06, 1.82); *p*=0.02; *n*=3644). Fathers with and EPDS score ≥13 at 18 weeks gestation did not have an increased risk of their child being diagnosed with anxiety (adj. OR 1.17 (0.63, 2.19); *p*=0.62; *n*=3275). Similarly, fathers with a CCEI-Anxiety subscale score >8 at 18 weeks gestation did not have an increased risk of their child being diagnosed with anxiety (adj. OR 1.45 (0.89, 2.37); *p*=0.13; *n*=2822).

## 5 Discussion

### 5.1 Key findings

This study is the first to date to compare the effects of maternal and paternal mood during pregnancy and the association with anxiety disorders in offspring at 18 years of age, as they move towards adulthood. The findings consistently demonstrate an increase in the risk of anxiety disorders in adolescent offspring after exposure to maternal depression in utero. In contrast, there is mixed evidence of an increase in the risk of anxiety disorders after exposure to maternal anxiety, and no evidence of an increased risk following exposure to paternal depression or paternal anxiety during pregnancy. However, when considering offspring anxiety with co-morbid depression both maternal antenatal anxiety and depression were associated with an increase in risk, whereas antenatal paternal mood was not. These findings are compatible with the hypothesis that foetal programming may account, at least in part, for the association between in utero exposure to maternal antenatal depression and offspring anxiety disorder. Before considering these findings in the context of the current literature, it is important to consider the strengths and limitations of the current study.

### 5.2 Strengths and limitations

A significant strength of this study is the use of a large population cohort, which is representative of families from the Bristol area of the UK. Another key strength is the use of the CIS-R, is a self-administered computerised interview, completed by the offspring to diagnose anxiety disorders, rather than relying on parental report or symptom scores of anxiety. This study also uses validated questionnaires to assess maternal and paternal depression and anxiety during pregnancy. Finally, this study uses prospective data collected over more than 18 years, yielding information on long-term potential effects of maternal stress in pregnancy.

However, there are a number of limitations to consider. In part due to the length of follow up, there was a relatively large attrition rate from recruitment during pregnancy to the assessment conducted with the offspring at 18 years of age. Previous studies have shown that the ALSPAC cohort attrition, like most population cohorts, is associated with socioeconomic disadvantage, leading to some limits on the generalisability across all sectors of the population (Boyd et al., 2012). In addition, it has been noted that mothers with mental health problems are less likely to continue participation in a longitudinal study (Bould et al., 2013). This attrition bias must be considered when generalising these findings to whole populations. A further limitation is that there is only one time point during pregnancy at which both maternal and paternal depression and anxiety levels were assessed (second trimester: 18 weeks); therefore we are unable to study possible timing effects during gestation. Other studies that used maternal scores of depression and anxiety at a later gestational age have shown similar associations with outcomes earlier in childhood (Austin et al., 2005; O'Connor et al., 2002; Sohr-Preston and Scaramella, 2006). Finally, there is substantial variability among children exposed to antenatal anxiety, which underscores the significance of postnatal or genetic factors (O'Donnell et al., 2014b).

### 5.3 Comparison with the current literature

A number of studies have shown that there is a strong association between maternal depression during pregnancy and increased rates of behavioural and emotional problems in their children (O’Connor et al., 2002; Van den Bergh et al., 2005; Weinstock, 2008). A smaller number of studies have shown that this association persists through into adolescence (O’Donnell et al., 2014b; Pawlby et al., 2011; Pearson et al., 2013a). In contrast, there are few studies that have explored the associations between paternal antenatal depression during their partner's pregnancy and later child outcomes (Kvalevaag et al., 2013; Ramchandani et al., 2008). Of particular interest is a study by van Batenburg-Eddes et al. (2013) that compares the effects of maternal and paternal depression and anxiety symptoms with offspring attention problems in two large cohorts. This study presents conflicting evidence on the effect of maternal antenatal depression and anxiety on the risk of attention problems in the offspring at 4 years of age, with one cohort showing an association and the other unable to replicate this finding. Neither cohort demonstrated an association between exposure to paternal antenatal depression or anxiety symptoms and adverse child outcome (Van Batenburg-Eddes et al., 2013). However, the authors only used one outcome measure in the children at 4 years of age, whereas there is evidence to suggest that there are several possible affected outcomes which can persist through into late adolescence (Glover, 2014; Pawlby et al., 2011; Pearson et al., 2013b).

The evidence for association between antenatal maternal anxiety and offspring anxiety was somewhat weaker than that for maternal depression. Further analysis of this finding with maternal antenatal anxiety showed that there is only attenuation after adjustment for postnatal maternal anxiety. However, this finding is in line with studies that have examined the effect of life-long maternal anxiety on offspring anxiety. Highlighted in a report by Schreier et al. (2008), there is an increase in the risk of anxiety in the offspring if their mother had a medical history of an anxiety disorder rather than antenatal anxiety specifically. This report also presents evidence to suggest that the risk of offspring anxiety is elevated after exposure to maternal social phobia or generalised anxiety disorder (Schreier et al., 2008). Therefore, it may be that the type of maternal anxiety disorder may contribute to mother–offspring aggregation of anxiety. Within our study, we were not able to assess specific parental anxiety disorders.

It has been postulated that an approach for strengthening evidence for causality regarding antenatal risk factors for childhood behavioural and psychiatric disorders is through the comparison of maternal and paternal symptoms during pregnancy (Lewis et al., 2013). The difference this study demonstrates between the effects of maternal and paternal depressive symptoms on child outcomes adds evidence to the case for a causal relationship of maternal antenatal depression on offspring development. Animal studies are suggestive and supportive of a biological mechanism to account for the observed changes in the offspring (Bowman et al., 2004; Coe et al., 2003; Kapoor and Matthews, 2005; Louvart et al., 2005). However, most of these studies are conducted in non-primate species making extrapolation of these studies to humans difficult (Beydoun and Saftlas, 2008). More substantive evidence of causality would come from experimental studies; however there are very few human studies of treatment intervention for maternal anxiety or depression in pregnancy. Extrapolation of the results from this study may infer that by providing support to women suffering from anxiety or depression antenatally we may improve outcomes for the mother and also reduce the risks of anxiety in the offspring.

We are only just starting to understand the potential mechanisms by which maternal mood in pregnancy may affect foetal and child development. There is evidence that if the mother is depressed or anxious the function of her placenta may be altered, with a down regulation of the enzymes 11β hydroxysteroid dehydrogenase 2, which metabolises cortisol (O’Donnell et al., 2012), and of monoamine oxidase A (Blakeley et al., 2013) which metabolises serotonin. These changes may allow more cortisol and serotonin to pass through the placenta, with the potential to influence a number of aspects of foetal development. Whilst these specific biological pathways are unlikely to account for all of any transmissible risk from mother to foetus, they represent plausible candidates for greater study.

## 6 Conclusion

This study showed an increased risk of offspring anxiety at 18 years of age after exposure to maternal antenatal depression at 18 weeks gestation. This association was not seen following exposure to paternal depression. These findings highlight the differences between antenatal depression exposures in different parents. This adds to support for a foetal programming effect occurring during pregnancy, leading to potentially long-lasting effects on the anxiety state of offspring. The exact mechanisms require further study, but this study does highlight the potential opportunity for preventative intervention by optimising the mental health of mothers during pregnancy.

## Conflict of interest

The authors declare no conflict of interest.

## Funding sources

ALSPAC Study has been funded by the Wellcome Trust, the Medical Research Council and the Univeristy of Bristol. Lauren Capron was supported by Imperial College London through a PhD Studentship. This research was undertaken at Imperial College Academic Health Science Centre, which is supported by the National Institute for Health Research (NIHR) Biomedical Research Centres funding scheme. The views expressed are those of the authors and not necessarily those of the NHS, the NIHR or the Department of Health.The sponsors had no role in study design and no role in data collection, data analysis, data interpretation, or writing of the manuscript.

## Figures and Tables

**Table 1:** Comparisons of the demographic information in the whole dataset and the sub-set of participants who undertook the CIS-R at 18 years of age. (*Significant result after Bonferroni correction=*α*/*n*=0.05/20=0.002).

	Mean (SD)		
**Whole dataset**	**Completed CIS-R**	**Comparison (*p* value)**
Average maternal age (years)	27.45 (5.01)	29.26 (4.62)	*p*<0.01*
Sex of infant (percentage of males)	55.2%	43.8%	*p*<0.01*
Prenatal smoking (average no. of cigarettes a day)	2.18 (5.25)	1.11 (3.82)	*p*<0.01*
Prenatal alcohol use (total no. of units per week)	0.13 (1.31)	0.12 (1.00)	*p*=0.91
Gestation (weeks)	39.47 (1.73)	39.48 (1.67)	*p*=0.73
Parity (no. of previous children)	0.88 (1.05)	0.71 (0.86)	*p*<0.01*
Birth weight (kilograms)	3.37 (0.59)	3.41 (0.55)	*p*<0.01*
Maternal educational status (0=None, 1=CSE, 2=Vocational, 3=O-level, 4=A-level, 5=Degree)	2.79 (1.28)	3.36 (1.20)	*p*<0.01*
Maternal depression at 18 weeks gestation (EPDS score)	6.92 (4.81)	6.19 (4.50)	*p*<0.01*
Maternal anxiety at 18 weeks gestation (CCEI-Anxiety score)	4.93 (3.53)	4.53 (3.35)	*p*<0.01*
Paternal depression at 18 weeks gestation (EDPS score)	4.23 (3.94)	3.95 (3.71)	*p*<0.01*
Paternal anxiety 18 at weeks gestation (CCEI- Anxiety score)	2.91 (2.79)	2.97 (2.64)	*p*=0.29
Maternal depression at 8 weeks postpartum (EPDS score)	6.21 (4.90)	5.77 (4.55)	*p*<0.01*
Maternal anxiety at 8 weeks postpartum (CCEI-Anxiety score)	3.48 (3.40)	3.29 (3.20)	*p*<0.01*
Paternal depression at 8 weeks postpartum (EPDS score)	3.87 (3.97)	3.66 (3.65)	*p*=0.01
Paternal anxiety at 8 weeks postpartum (CCEI-Anxiety score)	2.54 (2.69)	2.48 (2.51)	*p*=0.33

**Table 2:** Associations between maternal and paternal depression and anxiety and the offspring with the pure anxiety^a^ phenotype after adjustment for confounding variables and postnatal effects.

Risk	Percentage of offspring with anxiety disorder in exposed group (%)	Percentage of offspring with anxiety disorder in not exposed group (%)	Unadjusted odds ratio (CI's)	Fully adjusted odds ratio (CI's)^b^
Maternal Depression at 18 weeks gestation	11.1	6.2	1.71 (1.20, 2.44)	1.75 (1.19, 2.58)
(*n*=3599)	(*n*=3395)
Paternal Depression at 18 weeks gestation	7.6	6.7	1.14 (0.55, 2.38)	1.12 (0.51, 2.48)
(*n*=3187)	(*n*=3025)
Maternal Anxiety at 18 weeks gestation	9.2	6.6	1.43 (1.06, 1.94)	1.26 (0.89, 1.78)
(*n*=3537)	(*n*=3361)
Paternal Anxiety at 18 weeks gestation	8.9	6.6	1.39 (0.85, 2.27)	1.42 (0.79, 2.54)
(*n*=3181)	(*n*=3018)

a Pure anxiety phenotype included offspring who score for a primary or secondary anxiety disorder in the CIS-R excluding those who scored for comorbid depression.

b These results are adjusted for maternal age, gestational age, birth weight, parity, maternal alcohol consumption during pregnancy, maternal smoking during pregnancy, maternal educational status and postnatal parental depression and anxiety (measured at 8 weeks postpartum).
